# Hindering the biofilm of microbial pathogens and cancer cell lines development using silver nanoparticles synthesized by epidermal mucus proteins from *Clarias gariepinus*

**DOI:** 10.1186/s12896-024-00852-7

**Published:** 2024-05-04

**Authors:** Ahmed N. Alabssawy, Mohammed Abu-Elghait, Ahmad M. Azab, Hassan M. M. Khalaf-Allah, Abdelrahman S. Ashry, Ahmed O. M. Ali, Abu-Bakr A. A. Sabra, Salem S. Salem

**Affiliations:** 1 Zoology Department, Marine Science and Fishes Branch, Faculty of Science, Al-Azhar University, Nasr City, Cairo 11884 Egypt; 2 Botany and Microbiology Department, Faculty of Science, Al-Azhar University, Nasr City, Cairo 11884 Egypt

**Keywords:** Biosynthesis, *Clarias gariepinus* catfish, AgNPs, Antimicrobial, Antibiofilm, Cytotoxicity

## Abstract

Scientists know very little about the mechanisms underlying fish skin mucus, despite the fact that it is a component of the immune system. Fish skin mucus is an important component of defence against invasive infections. Recently, Fish skin and its mucus are gaining interest among immunologists. Characterization was done on the obtained silver nanoparticles Ag combined with *Clarias gariepinus* catfish epidermal mucus proteins (EMP-Ag-NPs) through UV–vis, FTIR, XRD, TEM, and SEM. Ag-NPs ranged in size from 4 to 20 nm, spherical in form and the angles were 38.10°, 44.20°, 64.40°, and 77.20°, Where wavelength change after formation of EMP-Ag-NPs as indicate of dark brown, the broad band recorded at wavelength at 391 nm. Additionally, the antimicrobial, antibiofilm and anticancer activities of EMP-Ag-NPs was assessed. The present results demonstrate high activity against unicellular fungi *C. albicans*, followed by *E. faecalis*. Antibiofilm results showed strong activity against both *S. aureus* and *P. aeruginosa* pathogens in a dose-dependent manner, without affecting planktonic cell growth. Also, cytotoxicity effect was investigated against normal cells (Vero), breast cancer cells (Mcf7) and hepatic carcinoma (HepG2) cell lines at concentrations (200–6.25 µg/mL) and current results showed highly anticancer effect of Ag-NPs at concentrations 100, 5 and 25 µg/mL exhibited rounding, shrinkage, deformation and granulation of Mcf7 and HepG2 with IC50 19.34 and 31.16 µg/mL respectively while Vero cells appeared rounded at concentration 50 µg/mL and normal shape at concentration 25, 12.5 and 6.25 µg/ml with IC50 35.85 µg/mL. This study evidence the potential efficacy of biologically generated Ag-NPs as a substitute medicinal agent against harmful microorganisms. Furthermore, it highlights their inhibitory effect on cancer cell lines.

## Introduction

Due to the constantly rising multidrug resistance, the effectiveness of antibiotics in treating bacterial infections has significantly diminished [1, 2]. It is estimated to cause over 700,000 deaths annually, rising to 10 million by 2050 if alternative solutions are not developed [3]. The efforts of scientists have been directed, in the last decades, to investigate new and effective therapeutic agents rather than the traditional antibiotics [4, 5]. These agents targeting different microbial mechanisms to avoid the resistance process which induced by targeting vital parts in the microbes such as cell wall, cell membrane, and DNA. Biofilm is one of these mechanisms which is considered the protective polymeric matrix embedding the bacterial cells and prevent antibiotic penetration [6].

Metal nanoparticles are incredibly useful in many industries, including electronics, purifying of water, medicine, and several biotechnologies [7–13]. Numerous procedures have been developed for the synthesis of NPs due to the large variety of uses afforded by these materials in various branches of research and industry [14–18]. Silver (Ag) NPs have received the most attention among the NPs now being used because of their distinctive properties, such as antimicrobial, antiviral, antifungal, anti-inflammatory, and anticancer activities [19–22].

Numerous studies have demonstrated the efficacy of Ag-NPs as a therapeutic agent for the treatment of infectious disorders, and they have also been included into the fabric of clothing during production to act as an antibacterial agent [23, 24]. Ag NPs are increasingly frequently used nanomaterials in the healthcare industry, and their annual global production is thought to exceed 500 tones. A crucial element of fish innate immunity is fish mucus, a byproduct of fish. This mucus serves as the fish skin’s natural defense barrier, continuously preventing the steady colonization of most infectious microorganisms including bacteria and fungi [25, 26].

Epidermal goblet cells create fish mucus, which is made up of mucins and other proteins, lipids, immunoglobulins, inorganic salts, and proteins floating in water that give it its distinctive lubricating qualities [27]. Some fish species, such as catfish of the Clarias genus, have long been used in traditional medicine to treat wounds, burns and tumors [28, 29].

Although nowadays many investigators have discovered substitute chemo-therapic methods for the purpose of bacterial and fungal infection. So, innate antimicrobial protein or peptide is one approach to fight off infection, cancer, and other diseases. The purpose of this work was to biosynthesize AgNPs utilizing the green and environmentally friendly approach of *Clarias gariepinus* catfish epidermal mucus proteins for the first time. Additionally, to thoroughly characterize EMP-AgNPs using various methodologies and assess their antibacterial, antibiofilm, and anticancer activities.

## Materials and methods

### Fish collection and maintenance

Catfish *Clarias gariepinus* specimens in growth were procured from a commercial fish farm with no prior history of contamination. Between 7:00 and 8:00 am, the fish were caught using a seine and taken right away to the Aquatic Culture Lab in the Animal House Building of the Zoology Department, Faculty of Science, Al-Azhar University. Average length (total length) 23–30 ± 0.5 cm and average weight of fishes150-220 ± 0.5 g. The five round plastic tanks held the fish (300 L). Half of the water in the tank was changed on alternate days to maintain sanitary standards and water quality. Fish were regularly checked for health, and any dead fish or fish with lesions were removed from the tank. They were kept in these tanks for 14 days so they could get used to the lab environment. The fish were given commercial catfish feed twice daily at 4% of their body weight, which contained 46% crude protein.

### Surgery and experimental protocol

The fish were removed from tanks and moved to an aquarium (100 L) with fresh water and aeration in the laboratory after 2 weeks of acclimatization. Three indoor tanks (100 L) with running water (23–25 °C) each held ten mixed-sex catfish *C. gariepinus*. Water characteristics included dissolved oxygen (5.5 to 7.0 mg/L), pH (7.10 ± 0.3), hardness and alkalinity (45 ± 5 mg CaCO_3_/L), and total ammonia (< 0.7 mg/L), all of which were within the species’ acceptable range. For wounding, fish were captured, anesthetized with Clove oil as 10 ml/L^−1^ then a tissue samples within 20 mm in diameter of muscle and skin (0.5 mm in depth) was lesion from the dorsal region near the central fin, in both sides (1punch in each side) by means of a biopsy punch. The wounds areas were measured by using the graduating ruler. These fish were used to gather mucus after 4 days.

### Skin mucus collection and preservation

The fish were starved for 10 h before the epidermal mucus could be collected. Individual fish were netted and moved to a 20 L aquarium containing anesthetic solutions. Using a sterile plastic spatula, mucus was carefully scraped from the dorsal body surface. In order to prevent sperm and intestine contamination, ventral cutaneous mucus was not collected. To avoid any external bacterial contamination, the skin mucus was extracted, immediately frozen, lyophilized, and kept at -24 °C until it was needed.

### Preparation of the mucus extract

Centrifuging mucus at 5000 RPM for 5 min after thoroughly mixing it with an equivalent amount of sterile physiological saline (0.85% NaCl) [30] and kept at -24 °C for future research.

### Biosynthesis of EMP- Ag-NPs

The following synthesis procedure was used to create green EMP-Ag-NPs. EMP- Ag-NPs are typically made by adding 10 ml of mucus extract to 50 ml of 1 mM AgNO3 water solution while vigorously stirring. Following that, the reaction will be finished in 6 h with the appearance of a brownish color in the reaction mixture, which will result in the creation of EMP- Ag-NPs.

### Characterization of biosynthesized EMP- Ag-NPs

The first thing that stands out is the colour shift. Initially, the extract was bright-yellow before being treated with AgNO3, but it changed to a brownish colour subsequently. Formation of MEP-Ag-NPs was analyzed by UV-Vis spectrometry (Shimadzu UV-1700, Japan) in the wavelength range of 200–800 nm. MEP-Ag-NPs reduction, stabilisation, and capping were all accomplished by a functional group that was detected by FTIR. Using potassium bromide to transform into a fine powder, the FTIR (Agilent system Cary 630 FT-IR model) analysis operates in the 500–4000 cm1 range. The Seifert 3003TT X-ray diffractometer, which used Cu K radiation (with a wavelength of 0.1546 nm), detected crystalline metallic silver. The size and shape of synthesised MEP-Ag-NPs were determined using TEM. A 120 kV acceleration voltage is used by the (TEM) on a ((JEM-1230(JEOL, Japan)) equipment. A copper grid that had been coated with carbon was used in the technique, which involved dropping a drop of colloidal solution containing bio-synthesized MEP-Ag-NPs onto the grid and loading the grid into a specimen holder. The elemental structure of MEP-Ag-NPs that have been bio-synthesised and their surface shape were both determined using SEM. The gadget (Japanese manufacturer JEOL, JSM-6360LA type).

### Biological assays

#### Culture media and microbial strains

The tested MEP-Ag-NPs were tested for antimicrobial activity against microbial strains such as Gram-positive bacteria (*Staphylococcus aureus* ATCC 25,923, and *Enterococcus faecalis* ATCC 29,212), Gram-negative bacteria (*Pseudomonas aeruginosa* ATCC 27,853, *Klebsiella pneumoniae* ATCC 13,883), and yeast (*Candida albicans* ATCC 10,213). Muller Hinton agar (MHA) and Tryptic soy agar (TSA) are the media used for bacteria and fungus, respectively. Antibiofilm of MEP-Ag-NPs was tested using biofilm forming *Staphylococcus aureus* ATCC 29,213 and *Pseudomonas aeruginosa* ATCC 9027 strains.

### Antimicrobial and minimum inhibitory concentration assays

Agar well diffusion technique was employed to determine MEP-Ag-NPs antibacterial properties in a preliminary manner. With minor changes from the prior work [31], the antibacterial activity of the MEP-Ag-NPs against Gram negative, Gram positive, and fungi was tested. Briefly, Phosphate buffer saline (PBS) was used to create microbial suspensions with an optical density of 0.5 McFarland standard. Prepared, autoclaved, and solidified MHA and TSA media were then injected with suspensions of bacterial and fungal species, respectively. The cork borer was then sterilised and used to drill 6 mm holes into the agar. Each hole was filled with 100 µl MEP-Ag-NPs, which were aseptically placed and stored at 4 °C for one hour. The positive controls for fungus and bacteria were fluconazole (25 µg/ml) and cefoxitin (35 µg/ml), respectively. After that, the plates were incubated for 24 h at 37 °C, after which the inhibitory zone diameters were measured. Moreover, a 96-well polystyrene plate with Mueller-Hinton broth for bacteria and tryptic soy broth for fungus was used to test the minimum inhibitory concentration (MIC) of MEP-Ag-NPs [32]. In each well containing broth and microbial suspensions, different quantities of MEP-Ag-NPs were dispersed. The plates were then incubated for 24 h at 37 °C. Using a microplate reader (Statfax, USA) at wavelength 600 nm, the MIC was established as the lowest concentration of MEP-Ag-NPs completely inhibiting bacterial or fungal growth [33]. As a control, medium lacking the investigated chemicals were employed. Micro-dilution plates (SPL, Korea) with and without active doses of MEP-Ag-NPs in 1% DMSO (final conc in the well) were inoculated with a 1: 100 dilution of overnight bacterial culture (inoculum size 10^6^ CFU/mL). The plates were subsequently incubated shaking at 100 rpm at 37 °C for 24 h. Data were analyzed in accordance with the recommendations of the Clinical Laboratory Standard Institute (CLSI) [34]. Three real duplicates for each experiment were accomplished.

### Antibiofilm assay

Bacterial strains were seeded in TSB containing 1% w/v glucose and incubated at 37 °C for 24 h to evaluate the biofilm inhibitory activity of Ag-NPs. The bacterial suspensions were then diluted to a turbidity which is equal to a 0.5 McFarland standard. Before adding the bacterial inoculum, 200 µL of TSB containing 1% w/v glucose was added to 96-well flat-bottom microtiter plates (MTP) from SPL, Korea. The MTP was treated with sub-inhibitory gradient doses of MEP-Ag-NPs, which were then incubated at 37 °C for 24 h. After incubation, the contents of the MTP wells were withdrawn, twice washed in PBS (pH 7.4), fixed for 10 min in 99% methanol, and stained for 20 min at 37 °C with 200 L of 0.1% w/v crystal violet (CV). Extra CV was eliminated, the plate cleaned with sterile distilled water, and room temperature drying was allowed for the plate. The dye was dissolved in 200µ L of 30% acetic acid, which was then applied to each well to quantitatively measure the biofilm inhibition. A microplate reader (Statfax, USA) was used to read the optical density O.D. at 492 nm. At a minimum, three duplicates of each experiment were performed. The formula used to get the percentage of inhibition was:% Inhibition = (OD growth - OD sample/OD growth control) x 100 [35, 36].

### Determination of cytotoxicity

Using the MTT method, the cytotoxic impact of the investigated MEP-Ag-NPs was assessed in breast cancer cells (McF7 ATCC HBT-22), hepato­cellular carcinoma cells (HepG2 ATCC HB-8065), and normal cells (Vero ATCC CCL-81) cell lines from VACSERA, Cairo, Egypt. In the 96 ­well tissue culture plate, 1 × 10^5^ cells/ml (100 µl) of cells developed a complete monolayer sheet after 24 h of incubation at 37 °C. The 96 well micro ­titer plates’ growth medium was decanted when a confluent sheet of cells had developed, and the cell monolayer had gone through two rounds of wash media washing. In 2% serum containing RPMI medium (maintenance medium), the test material was diluted twice. Three wells were used as controls, receiving just maintenance medium, while the other wells received 0.1 ml of each dilution for testing. After an incubation period of 37 °C, the plate was examined. Cells were analyzed to determine the physical manifestations of toxicity, including partial or complete loss of the monolayer, rounding, distortion, or cell granulation. The MTT solution (5 mg/ml in PBS) was made (BIO BASIC CANADA INC.). To each well, a 20 µl MTT solution was added. For five minutes, shake at 150 rpm to properly blend the MTT into the medium. Allow the MTT to metabolize for 4 h in an incubator set at 37 °C with 5% CO2. Discard the media. (If required, dry the dish using paper towels to get rid of the residue. Formazan (MTT metabolic product) suspension in 200ul DMSO. For five minutes, shake at 150 rpm to properly combine the formazan and solvent. At 560 nm, read optical density, and at 620 nm, remove background [37]. There should be a direct relationship between optical density and cell number [38].

### Result and discussion

A reliable chemical or physical barrier against invasive infections is provided by the fish skin mucus (Table 1). A slimy, slick film known as mucus covers the epithelial surfaces in fish. It is also referred to as a viscous colloid that includes mucins, which are antibacterial enzymes, proteins, and water. In two ways, it contributes significantly to the innate immune system. First of all, by continuously producing and regularly shedding, it inhibits the adherence of pathogens, persistent colonization of potentially pathogenic bacteria, and invasion of parasites [39]. Second, it includes a variety of components that contribute to innate immunity, including proteins and enzymes like lysozyme, proteolytic enzymes, transferring, alkaline phosphatase, and variety of antibacterial proteins and peptides, etc [39, 40].

Due to the high water (moisture) content and the presence of gel-forming macromolecules, epidermal mucus of catfish species showed higher amounts of moisture ranging from 88 to 90% [41]. According to a previous study, the lipid content of mucus from some fish species was up to 20 times higher per unit area than that of human sebum. These free fatty acids may act as antioxidants and provide defense against bacterial and fungal attack [42].

**Table 1:** Protein, fat, carbohydrate, moisture and ash contents of *Clarias gariepinus* epidermal mucus

Protein	Fat	Carbohydrate	Moisture	Ash
6.35 ± 1.72	0.57 ± 0.06	2.24 ± 1.31	92.04 ± 2.43	1.67 ± 1.02

### Biosynthesis of MEP-Ag-NPs

**Fig. 1:**
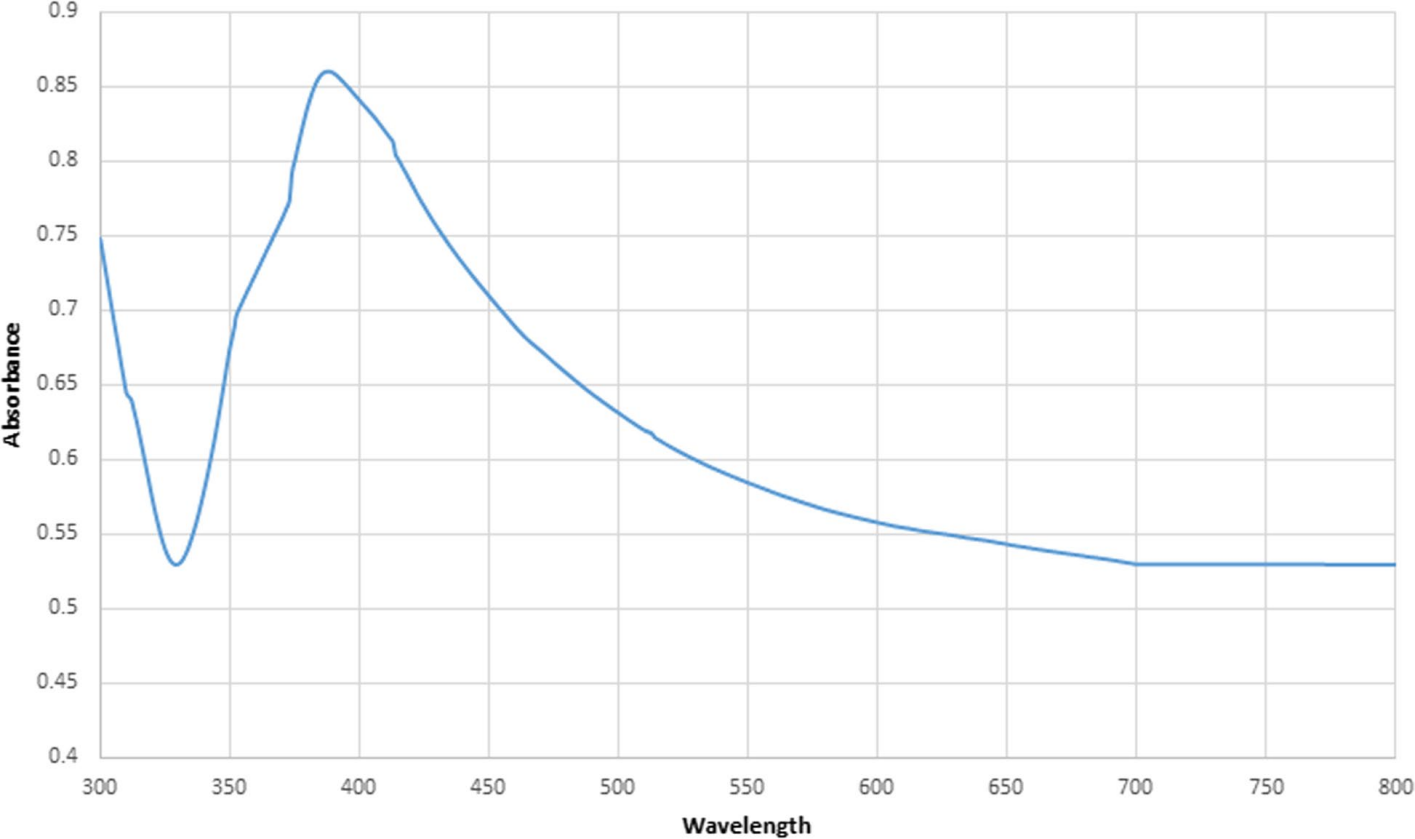
Absorption peaks of Ag-NPs using a UV-Vis Spectrophotometer

In this study, when mixed AgNO_3_ with mucus extract, reduction to formation MEP-Ag-NPs within 6 h, where appear from light yellow to dark brown as indicate successfully synthesized of MEP-Ag-NPs. Other studies that concur with this study show that the colours were altered from yellow to dark brown [43]. Surface plasmon resonance (SPR) is associated to the colour shift to deep dark brown. The UV-Vis spectrophotometer measure wave length between 200 and 800 nm. Where wave length change after formation of MEP-Ag-NPs as indicate of dark brown, the broad band recorded at wave length at 391 nm Fig. 1.

**Fig. 2:**
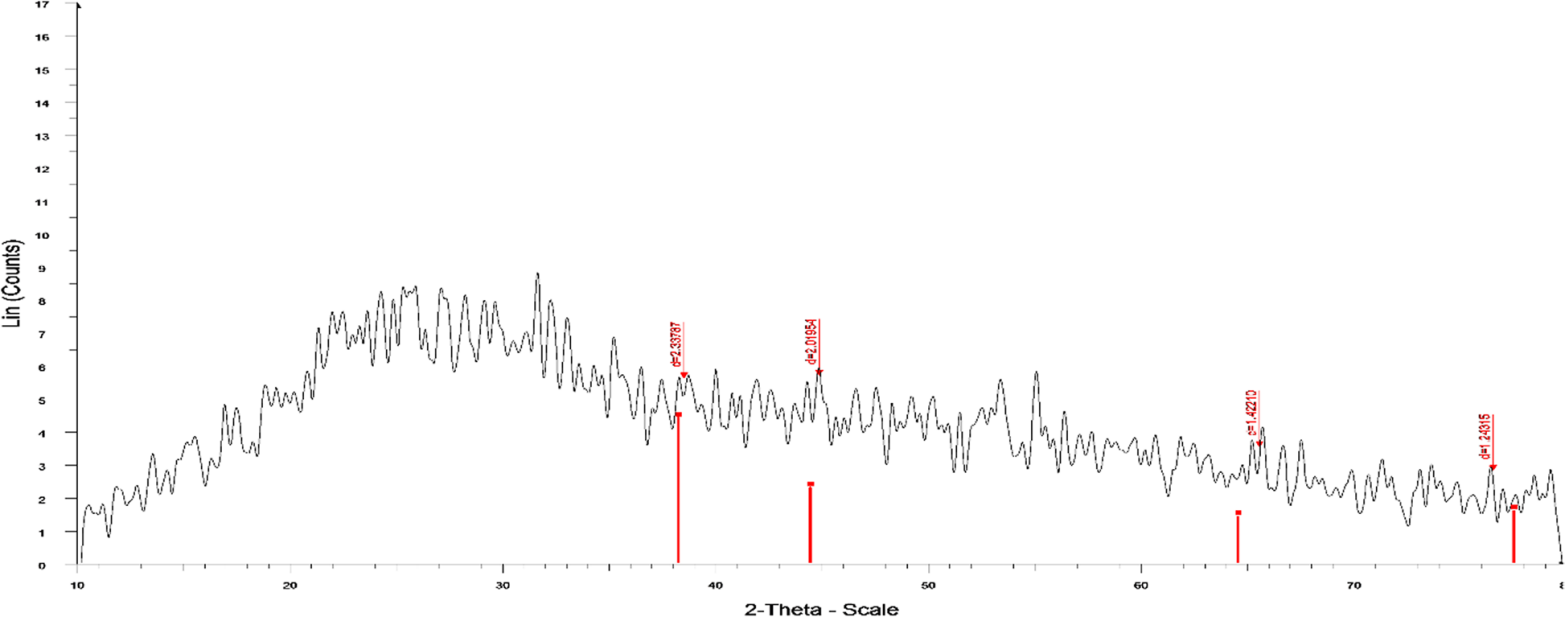
XRD pattern for MEP-Ag-NPs

Every crystalline substance has a unique diffraction, which is determined by X-ray diffraction (XRD), which is also used to detect crystalline structure and nanoparticle shape. The output was shown in Fig. 2. Four main diffraction peaks at 2 values of 38.1, 44.2°, 64.4, and 77.2°, which are connected to reflection planes of (111), 200), (220, and (311, respectively, are shown in the XRD image of biosynthesized MEP-Ag-NPs. This study is corresponding to other study [44].

**Fig. 3:**
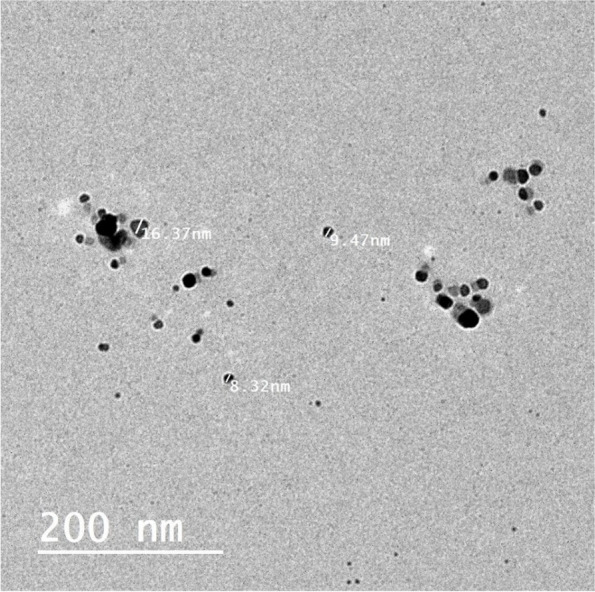
TEM micrograph of MEP-Ag-NPs

**Fig. 4:**
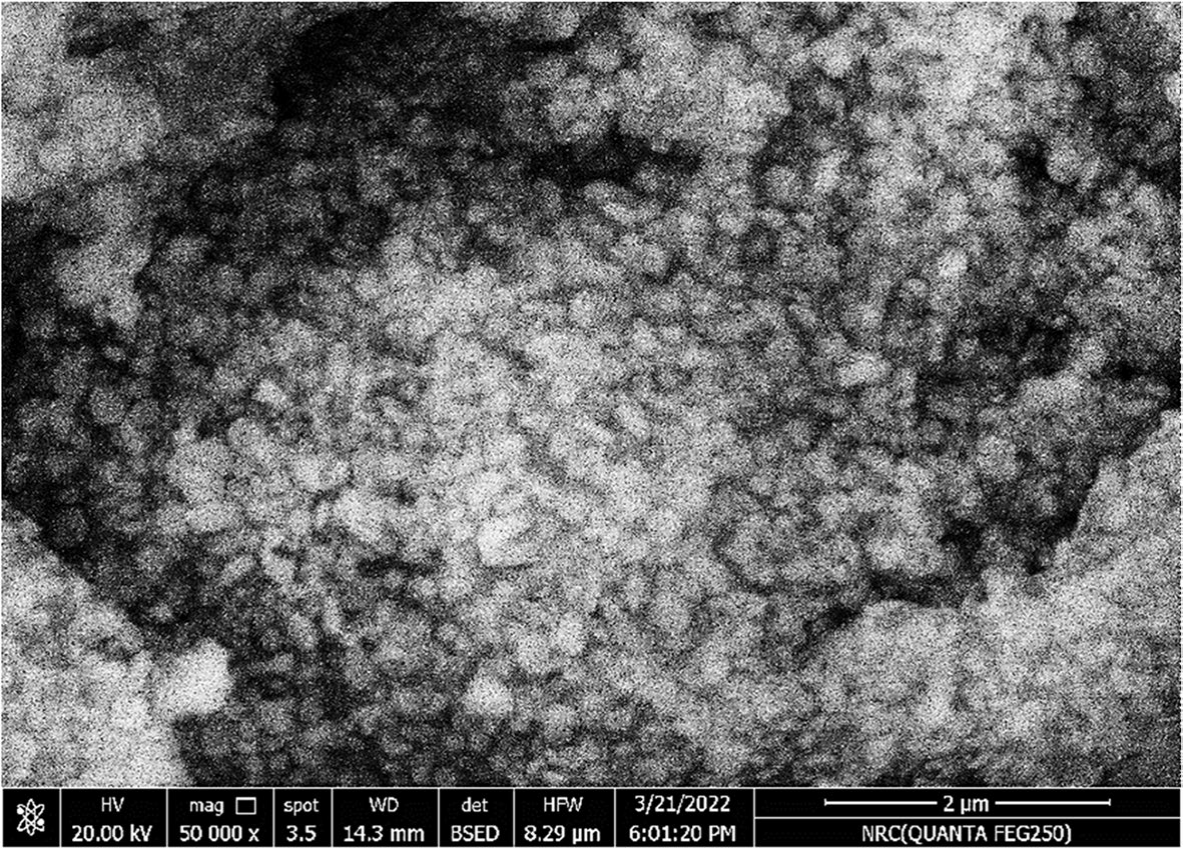
SEM micrograph of MEP-Ag-NPs

To evaluate morphology and average size, TEM and SEM were utilized. Figure 3 from a TEM image shows the existence of polydisperse, spherical MEP-Ag-NPs with sizes ranging from 4 nm to 20 nm. The range of Ag-NPs reported in the earlier publication ranged from 37 to 87 nm [44]. The development of spherical MEP-Ag-NPs is seen in Fig. 4 of the SEM micrograph analysis. The paper mentioned above shows how mucus extract may produce polycrystalline, spherical, uniform, and stable MEP-Ag-NPs. These studies which agreement with the bio-reduced Ag-NPs that have been mentioned previously [19, 22, 45].

**Fig. 5:**
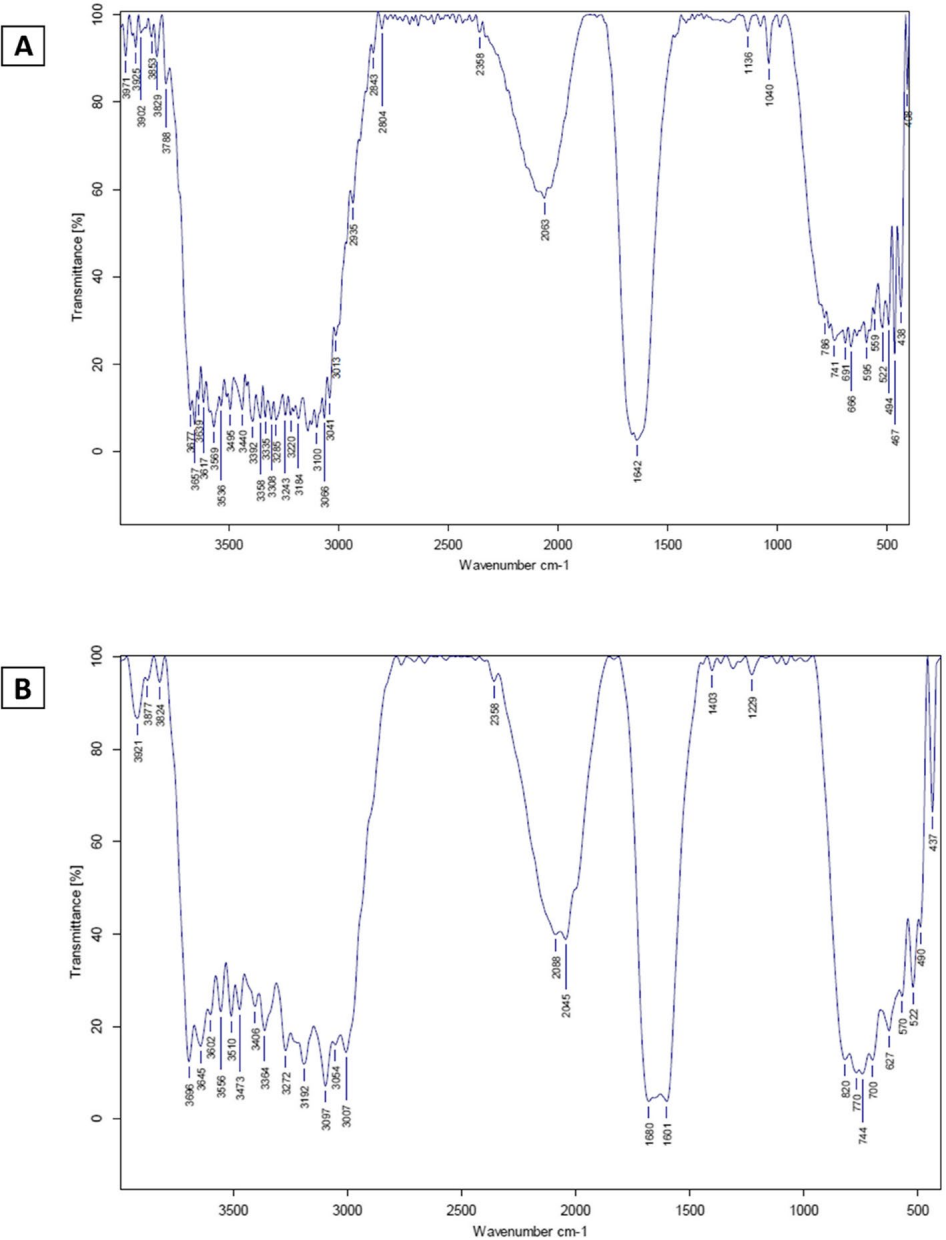
FTIR analysis of epidermal mucus extract (**A**) and MEP-Ag-NPs (**B**)

As seen in Fig. 5A and B, distinct functional groups of MEP-Ag-NPs were identified by FTIR. The FTIR analysis was performed to determine how Ag and metabolites of the epidermal mucus extract interacted, resulting in intermediate capping and reduction for evenly dispersed Ag-NPs in their colloidal solution. As a result of the interaction between MEP-Ag-NPs and the (OH) group, the FTIR analysis of MEP-Ag-NPs produced a number of bands that indicate wide bands from 540 to 775 cm1. Bands that signify the carboxyl group of the C-C stretching vibration and the amide I bands of proteins also emerge at 1642 cm1. In addition, a band can be seen at 2063 cm 1, which indicates that it reflects the stretching vibrations of CHx. The peaks located at 3900 –3000 cm − 1 were identified by the N-H groups or O-H vibration stretching. From previous result the extract which detected from spectrum, where interacted with silver ions to form MEP-Ag-NPs. This result confirms the mucus extract has the predominant role for bio-reduction of Ag.

### Antimicrobial activity

**Fig. 6:**
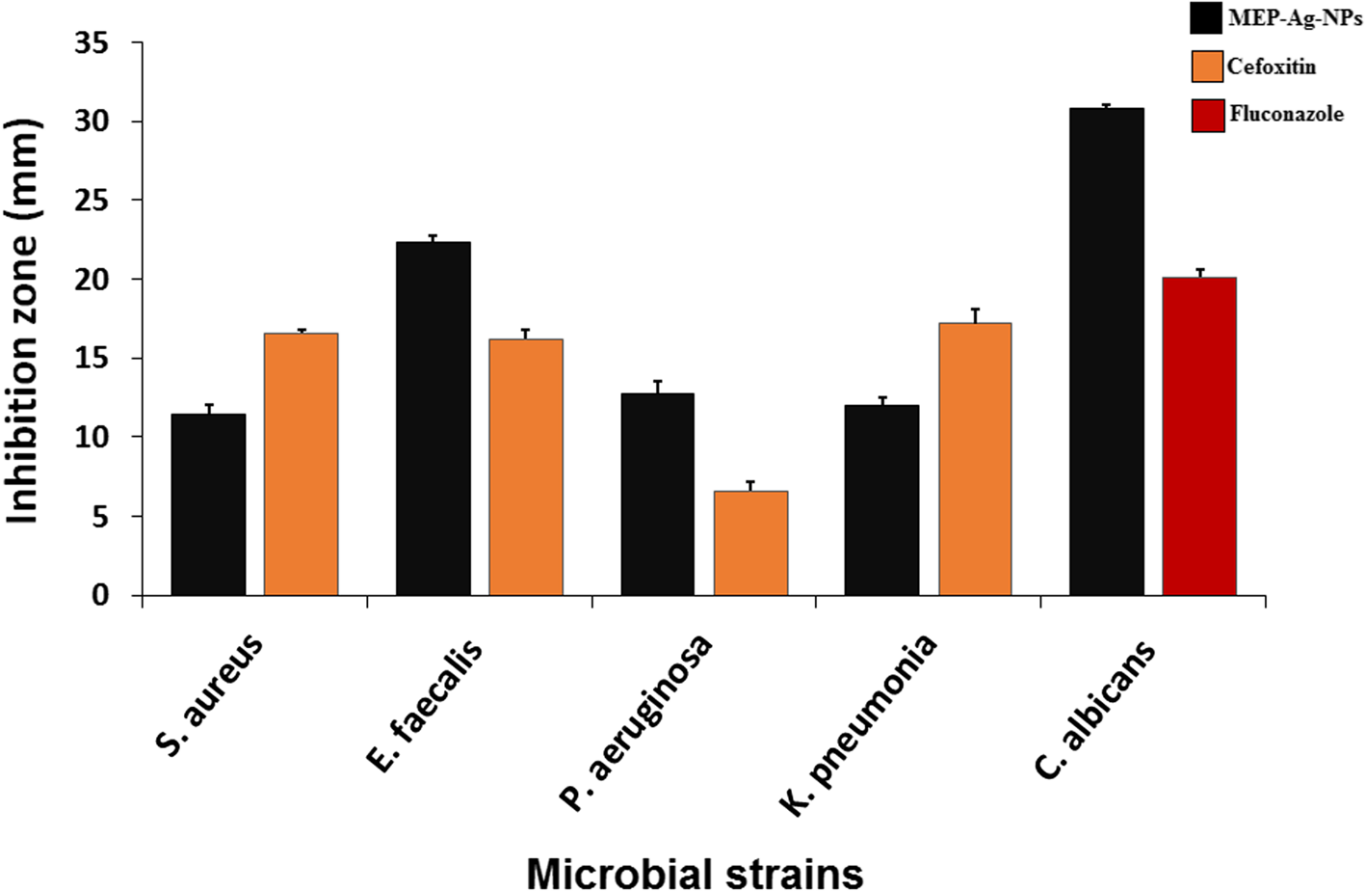
The antimicrobial activity of MEP-Ag-NPs against different microbial strains

According to the world health organization reports, microbial antibiotic resistance has been considered a global problem in the last decades because of the rapidly spreading although the appearance of new synthetic derivatives to the traditional antibiotics. Therefore, there is a mandatory need to investigate a new and alternative therapeutic agent, natural or synthetic, to solve this problem. Nanoparticles are one of the recently emerged agents used by scientists in several disciplines and have largest share of their efforts in the field of combating multidrug resistant microbes [46–49]. The antimicrobial activity screening of MEP-Ag-NPs was investigated against Gram positive, Gram-negative bacteria in addition to unicellular fungi. The result showed variable activities regarding to different organisms since it showed antimicrobial activity against all microbes with different degrees, but the strongest activity was showed against unicellular fungi *C. albicans* with inhibition zone 30.8 ± 0.24 mm followed by *E. faecalis* with inhibition zone 22.36 ± 0.41 (Fig. 6).

### Antibiofilm activity

**Fig. 7:**
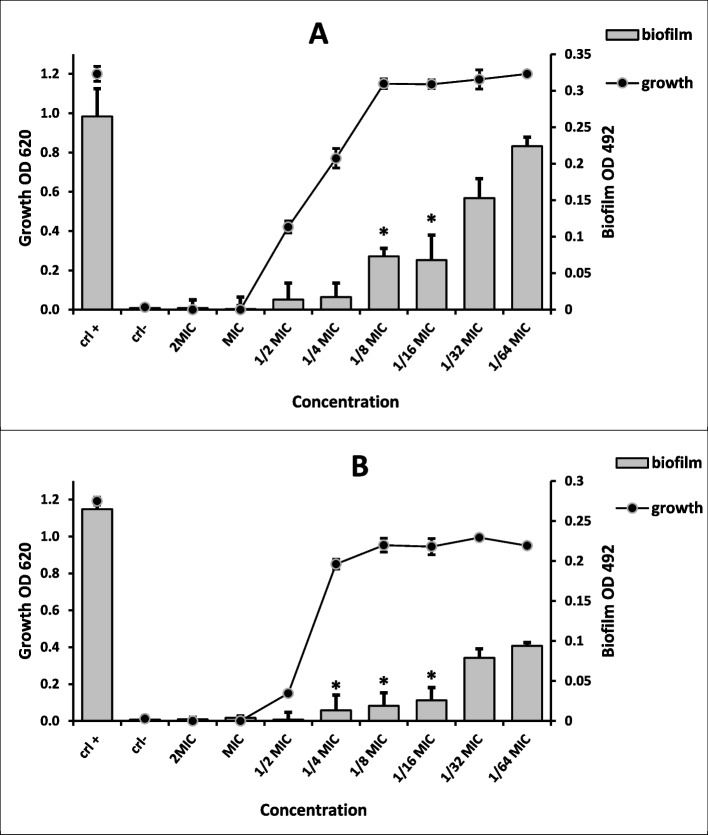
The antibiofilm activity data of MEP-Ag-NPs against the *S. aureus* (**A**) and *P. aeruginosa* (**B**) strains

Antivirulence strategy is one of the most effective methods to combat the antibiotic resistance phenomenon. This strategy depending on creating a new target for the antimicrobial agents rather than the microbial survival, which is the main reason motivate the bacteria for developing a resistance gene, but the target of antivirulence agents is the microbial virulence factors which considered its weapons that introduce the pathogenicity to the host [50, 51]. Biofilm is a complex polymer matrix produced by the population of microbes to protect itself from the host immune system and antibiotics moreover exchange water and nutrients between each other within this matrix. Biofilm is one of the important virulence tool that might be a suitable and effective target for the antivirulence agents [52, 53]. In the present study, after evaluating the antibacterial activity of MEP-Ag-NPs which exhibited antimicrobial activity against the planktonic bacterial growth of the Gram-positive *S. aureus* and Gram-negative *P. aeruginosa*, the MEP-Ag-NPs was tested at sub inhibitory concentrations against biofilm formation of the previous mentioned strong biofilm producing pathogens. The result showed strong antibiofilm activity against both *S. aureus* and *P. aeruginosa* pathogens where the concentrations 1/8, 1/16, 1/132 and 1/64 of MIC (0.125-0.062-0.031- 0.015 mg/ml) inhibit the biofilm formation of *S. aureus* by 74.33, 72.45, 42.26, and 15.47% respectively in a dose dependent manner without affecting planktonic cell growth. The most significant inhibition concentrations were 0.125 and 0.062 mg/ml (*p* < 0.05) as shown in (Fig. 7A) while 1/4, 1/8, 1/16, and 1/32 of MIC (2.5-1.25-0.62-0.31 mg/ml) inhibit biofilm formation of *P. aeruginosa* by 94.9, 92.83, 90.18, and 70.18% respectively in a dose dependent manner without affecting planktonic cell growth. The most significant inhibition concentrations were 1.25, 0.62 and 0.31 mg/ml (*p* < 0.05) as shown in (Fig. 7B). The result reflects the importance of MEP-Ag-NPs for elimination the biofilm formation in both Gram positive and Gram-negative bacteria which is recommended to be an effective alternative agent to antibiotics after further in vivo and clinical investigations which considered a step toward introducing a resolve to the antibiotic resistance phenomenon.

### Anticancer effect of the tested compound

**Fig. 8:**
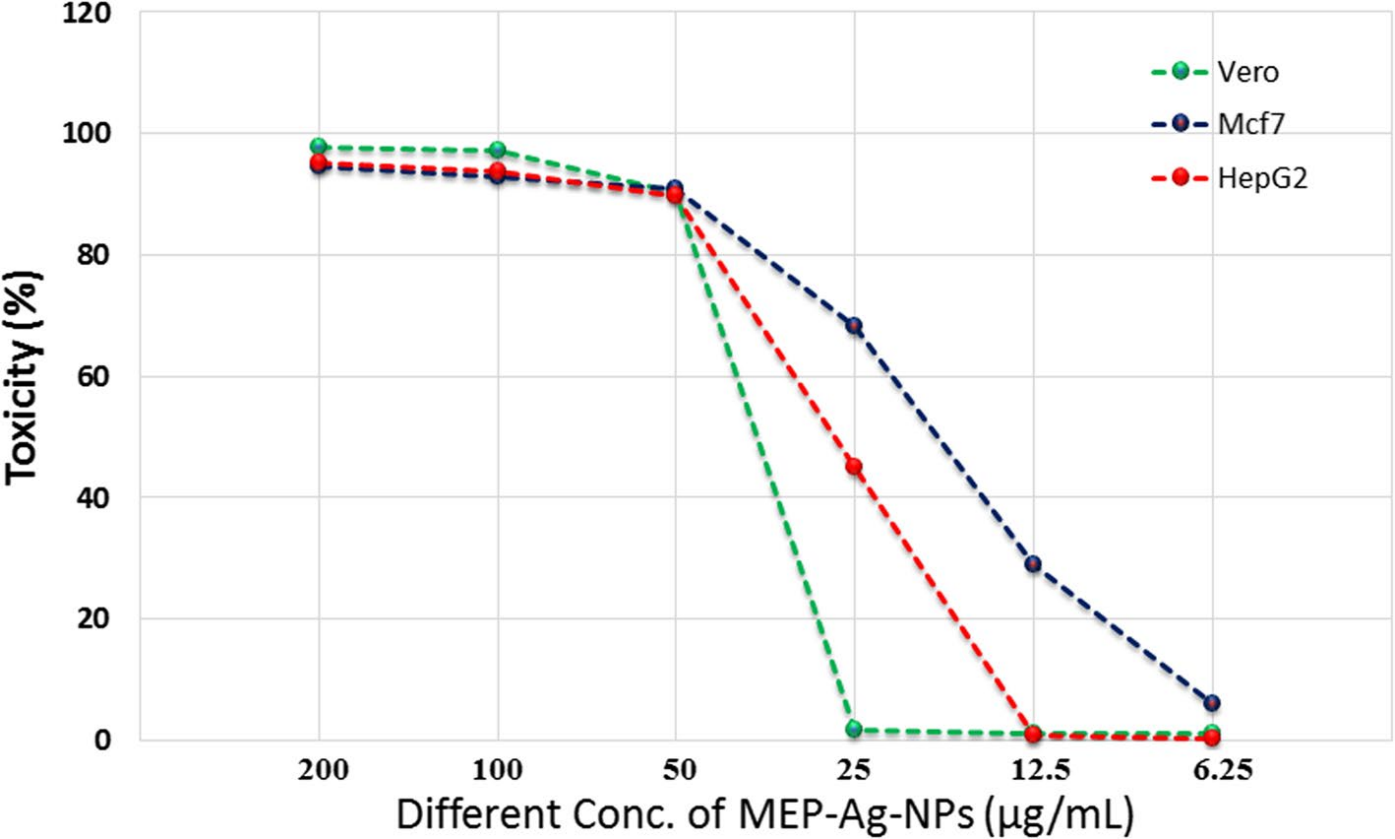
Effect of MEP-Ag-NPs on Vero, Mcf7, and HepG2 cells at different concentration

The cytopathic effect of MEP-Ag-NPs was investigated against normal cells (Vero), breast cancer cells (Mcf7) and hepatic carcinoma (HepG2) cell ­lines at concentrations (200–6.2 µg/mL). MEP-Ag-NPs exhibit complete detachment of monolayer cheat of Vero, Mcf7 and HepG2 cell lines at concentration 200 µg/mL (Fig. 8). MEP-Ag-NPs at concentrations 100, 5 and 25 µg/mL exhibited rounding, shrinkage, deformation and granulation of Mcf7 and HepG2 with IC50 19.34 and 31.16 µg/mL respectively, while Vero cells appeared rounded at concentration 50 µg/mL and normal shape at concentration 25,12.5 and 6.25 µg/ml with IC_50_ 35.85 µg/mL (Fig. 8). Comparable results demonstrate that biosynthesized nanoparticles made from cell-free extracts are more biocompatible, safer, and appropriate for use in biomedical applications including pathogens prevention, fabrics and dressings [23, 31, 32].

## Conclusion

In conclusion, this research investigated the antimicrobial activities of silver nanoparticles combined with epidermal mucus proteins isolated from the *Clarias gariepinus* catfish, which had significant activity opposed to *S. aureus* and *E. faecalis*, *P. aeruginosa* and *K. pneumoniae*, and the unicellular fungus *C. albicans*. Additionally, there was a notable great cytopathic effect against the breast cancer cells (MCF7) and hepatic carcinoma (HepG2) cell lines. The study also provides evidence that MEP-Ag-NPs have a perfect antibiofilm results against both *S. aureus* and *P. aeruginosa* pathogens. To identify and purify more novel nano silver composite Ag-NPs from various fish epidermal mucus proteins as natural sources of antimicrobial and anticancer, a thorough investigation is needed. Understanding these nano silver composite Ag-NPs’ modes of action will help scientists create new medications to treat various bacterial, fungal, and cancer diseases.

## Data Availability

No datasets were generated or analysed during the current study.
